# Wound Healing Effects of New Cream Formulations with Herbal Ingredients

**DOI:** 10.3390/pharmaceutics17070941

**Published:** 2025-07-21

**Authors:** Derya Algül, Ertuğrul Kılıç, Ferda Özkan, Yasemin Yağan Uzuner

**Affiliations:** 1 Department of Pharmaceutical Technology, Faculty of Pharmacy, Yeditepe University, 34755 Kayışdağı, Istanbul, Turkey; deryaalgul@gmail.com; 2 Department of Physiology, School of Medicine, Medeniyet University, 34700 Uskudar, Istanbul, Turkey; ertugrul.kilic@medeniyet.edu.tr; 3 Department of Breast Diseases and Breast Cancer, Istanbul Oncology Hospital, 34846 Maltepe, Istanbul, Turkey; ferda.ozkan@istanbulonko.com

**Keywords:** wound care, storax (*Liquidambar orientalis* Mill.) *Calendula officinalis*, St. John’s Wort, aescin

## Abstract

**Aim**: To prepare two different kinds of wound care creams containing plant extracts and examine their effectiveness in comparison with a placebo cream and a commercial wound care cream, Madecassol^®^. **Methods**: The two cream formulations were developed using the same placebo cream (PC) as base cream. One formulation contained balsam of oriental sweet gum, or Levant storax, named as Levant Storax Cream (LSC); the other contained oil of Calendula, extract of St. John’s Wort, aescin (an extract of horse chestnut), and freeze-dried powder from Aloe vera (L.) Burm. f. leaf juice, designated as Complex Cream (CC). In the characterization of the creams, organoleptic properties, pH, viscosity, size distribution, and zeta potential of oil globules were measured. Furthermore, the stability of the creams was assessed under different environmental conditions. In vitro studies were performed by using an excisional wound model in rats to assess the potential of the creams for stimulating wound healing. The efficacy of LSC and CC was compared with a commercial reference cream, Madecassol^®^ (M), and the placebo control. The study was also designed with a negative control group of rats that were not treated but handled the same way as the other treatment groups. The wound contraction rate, total skin thickness recovery, and results of histopathological parameter examinations were used to compare the effectiveness of the treatments. **Results:** The stability of formulated creams confirmed that they were stable for the duration of the study. In vivo studies showed that rats treated with LSC achieved the highest wound healing rates when compared with the other groups. A better response was recorded for the CC-treated population when compared to both control and placebo groups, but there was no significant difference seen in healing score between CC and M groups.

## 1. Introduction

A wound is defined as a disruption of the structure and functional processes of tissues, which can be caused by trauma, surgical intervention, pressure, burns, cuts, or a disease due to some underlying pathological condition [1]. Wound management is a chronic problem faced by humans since time immemorial, and clinical cases often report patient loss due to septicemia caused by wounds [2,3]. Over the last few decades, we have started to understand in detail the mechanisms of wound healing and the factors that influence them [4]. The wound healing process is a complex and dynamic process involving four related and overlapping phases: hemostasis, inflammation, proliferation, and maturation/remodeling [3,4]. Immediately after the injury, hemostasis occurs, with the release of von Willebrand factor from endothelial cells, resulting in aggregation, accumulation, and activation of platelets. These platelets subsequently release mediators that cause the formation of a fibrin clot, which occludes the wound and prevents blood loss [5]. Higher calcium levels cause contraction of smooth muscle, which narrows and reduces blood flow to blood vessels. This phase usually continues for only a few minutes [5]. On the other hand, the inflammatory phase characterizes histamine and serotonin release from mast cells, which induces vasodilation and attracts neutrophils and monocytes. Invading pathogens and debris are phagocytosed by these immune cells, and cytokines are released to initiate the next stage of healing; this period lasts between 0 and 3 days [5,6]. The next phase, the proliferative phase, lasts for about 3 to 12 days and is characterized by the formation of granulation tissue by fibroblasts, which facilitates the migration of keratinocytes. This stage also promotes angiogenesis. Macrophages and mast cells secrete growth factors important in re-epithelialization and vascular network regeneration [5,6]. The last step in the wound healing process is the maturation phase, where collagen remodeling and wound contraction occur [6,7]. Myofibroblasts, from fibroblasts, mediate this phase, and this process may take from 3 days to months. Type III collagen is replaced with type I collagen, leading to scar maturation and increased tensile strength [7]. These alterations are regulated by transforming growth factor-beta (TGF-beta), but it is recognized that some skin structures could not be completely regenerated, so that tensile strength will not exceed 80% of that of the initial skin [7].

Herbs have been used for centuries to promote wound healing. Although empirical use is centuries old, the scientific evidence for their efficacy and their active compounds has only recently been investigated by researchers and their effects have been published [8,9]. Natural compounds, with their anti-inflammatory, antimicrobial, antioxidant, or pro-angiogenic activities, can enhance the wound healing processes [10,11]. There are studies showing that *Calendula officinalis* acts by stimulating fibroblast activity and collagen deposition, and they corroborate the waterfalls of pro-inflammatory cytokines [12]. *Hypericum perforatum* (St. John’s Wort) is rich in flavonoids, and hyperforin can modulate inflammatory responses and exhibit potent antimicrobial activities [11,13,14]. *Aesculus hippocastanum* (Horse chestnut) contains an ingredient called aescin, which is a saponin known for its vascular protective and anti-edematous properties in promoting microcirculation and oxygenation of the tissues [15]. The polysaccharides in aloe vera also stimulate fibroblast proliferation and production of type I collagen [16]. In addition, Allantoin obtained from Symphytum officinale has the role of stimulating epithelial regeneration by cell proliferation [17,18,19]. Another potential product is *Liquidambar orientalis* (Levant storax), a local plant grown in the eastern Mediterranean regions of southwest Turkey, including Marmaris, Fethiye, Köyceğiz, Dalaman, and Urla [19,20]. Turkish sweet gum trees usually do not have balsam channels that secrete oil, but vertical balm channels are formed on both sides of the wounds after artificial or natural injury. The excrement is in the form of resinous exudate known as balsam and is obtained by pressing the barks in cold water and subsequently boiling them to collect the surface exudate [21,22]. Styrax liquidus was historically used in the process of mummification in ancient Egyptian civilization and has also been used for medicinal purposes in Turkish folk medicine, including an antiseptic for burns and wounds, for peptic ulcers, parasitic infections, and nocturnal enuresis, and as an expectorant [20,21]. Styrax is still consumed as a mixture with honey (in a 1:5 proportion) or in the form of pills composed of boiled seeds in thisregion [22]. Its anti-ulcerogenic potential was established in one study using an in vivo model [23]. In addition, other studies reported various activities, including antibacterial, antiviral, antifungal, antihypertensive, and anticonvulsant effects [24,25,26]. The ethnobotanical use of this plant has motivated scientists to investigate the composition and pharmacological properties of similar compounds and natural drugs that humans have exploited. The composition of Styrax has been investigated by various scientists [27]. The main components of the essential oil were determined as styrene (92.6%) and α-pinene (2.2%) [27,28]. Styrax has no genotoxicity risk and has been suggested as a protection against genotoxic agents [29]. In a previous study, Styrax liquidus was also shown to have cytotoxicity and increased apoptotic potential on the laryngeal cancer cell line, HEp-2, and human embryonic kidney cell (HEK-293), which was supplemented by a reduction in the population of HEp-2 laryngeal cancer cells and enhanced expression of apoptosis guide genes. These results indicate that Styrax liquidus could be a promising candidate for cancer therapy [30]. Other studies have suggested its effectiveness against prostate cancer [31] and human colorectal cancer cells [32]. However, current treatment options continue to heavily depend on synthetic agents, which either have poor biocompatibility, may irritate the tissue, or do not retain sufficient moisture in the wound bed, all of which are elements integral to achieving healing [33]. The growing aging population, in conjunction with increasing incidences of diabetes and obesity amongst younger individuals, has significantly augmented the risks of injuries, thus propelling the demand for quality wound care products. Whilst this demand poses significant financial challenges, it also presents tremendous growth opportunities for the global wound care products market [34]. Novel products incorporating natural polymers, stem cells, and mRNA highlight advances being made in that area [34]. Treating chronic wounds to achieve complete recovery is still a challenging task that possesses challenges as well as potential market opportunities [34,35]. The challenge of establishing stable, potent, bioactive topical herbal formulations remains a critical area of research. This study focuses on the development of two cream formulations based on a common placebo cream (PC). The formulations were composed of the balsam of oriental sweet gum (LSC), Calendula oil, St. John’s Wort extract, aescin, freeze-dried powder of Aloe vera (L.) Burm. f. leaf juice, and Complex Cream (CC). The creams were characterized by measuring their pH, viscosity, and zeta potential of the oil globules. In addition, the stability of the creams was monitored in different environmental conditions. The effectiveness of the creams as topical wound healing agents was assessed in an in vivo excisional wound model in rats. LSC and CC performances were compared with a commercial reference cream, Madecassol^®^ (M), and the PC. A negative control group of untreated rats was also included (C group). Wound contraction percentages, full skin thickness development percentages, and histopathological parameters were used to evaluate the efficacy of the different treatments.

## 2. Materials and Methods

### 2.1. Materials

(PC) was an O/W cream containing an aqueous phase and an oil phase containing the materials as listed in Table 1.

### 2.2. Preparation of the Cream Formulations

#### 2.2.1. Pre-Formulation Studies

To produce a stable base cream, pre-formulation studies were carried out in accordance with the guidelines from the literature and pre-formulation principles. Several formulations were tried, and after the organoleptic assessments and subjecting each candidate to stress stability tests, one of the formulations was selected. This formulation became the PC, which was used as the base formulation for the other two formulations. CC incorporated extracts of Calendula oil, St. John’s Wort extract, aescin, and freeze-dried powder from *Aloe vera* (L.) *Burm.f.* leaf juice, which were added at concentrations that would remain consistent within the ranges discussed in the literature and recommendations of the extract suppliers, while LSC contained balsam of oriental sweet gum.

#### 2.2.2. Preparation of the Creams

The aqueous phase was prepared by dissolving EDTA and glycerin in deionized water. The oil phase comprised shea butter, squalene, cetostearyl alcohol, cetearyl olivate, sorbitan olivate, petroleum jelly, and caprylic/capric triglyceride. Aqueous and oily phases were prepared and heated to 70 °C separately in a thermostated water bath. The aqueous phase was added slowly to the oil phase while mixing with a Silverson L4R homogenizer. The emulsion was homogenized at varying speeds until it was cooled down to 30 °C, and the remaining ingredients were added and homogenized to complete the production. Triplicate samples of each cream formulation were prepared by using the same method and were evaluated for their stability and efficacy.

### 2.3. Characterization Studies of the Creams

Each cream sample was evaluated organoleptically for appearance, color, odor, spreadability, and, after-feel on the skin. The pH (Seven Multi S47, Mettler-Toledo, Colombus, OH 43240, USA), viscosity (Brookfield Viscometer, Model- RVDII+, Ametek Brookfield, Middleborough, MA 02346, USA), conductivity (Seven Multi S47, Mettler Toledo, Colombus, OH 43240, USA), zeta potential (Zetasizer Nano-Zs, Malvern Panalytical Ltd., Malvern, WR141XZ, UK), mean size, and size distribution of the oil globules of the creams were measured microscopically [36]. Microscopic examination and microphotograph counts were made to determine the arithmetic mean particle size of 1000 particles and the size distribution [36] with the help of an image analysis system connected to the microscope (Leica CTR 6000 microscope fitted with a DFC 350 FXR2 camera equipped with its own software). Microbiological tests were conducted using a neutralizing solution for dilution and plating the products on nutrient media (SDA and TSA) to assess bacteria, molds, yeast growth, and potential pathogens [37].

#### Stability Tests

High speed centrifugation and freeze-thawing (5 cycles) were the initial stress testing used to detect the early instability problems during the selection of PC. Accelerated stability studies were performed with LSC and CC in triplicate and at three different storage conditions: (i) 25 ± 2 °C and 60 ± 5% relative humidity (RH), (ii) 4–8 °C (refrigerated conditions), and (iii) ambient room temperature in the dark. Evaluations were carried out on days 0, 7, 30, and 60. The samples were investigated organoleptically, and the viscosity, pH, zeta potential, and particle size of each batch of P, LSC, and CC were measured at predetermined times [36,37,38,39,40,41,42]. In addition, the stability of formulations was analyzed using a Formulaction™ Lab device at 40 °C for 2 days, during which the amount of transmitted and backscattered light was recorded at hourly intervals [36,43,44,45]. For these tests, 20 mL of the fresh emulsion was placed in a 43 mm high flat-bottomed glass cuvette, and the cuvette was scanned at 40 °C every hour. Emulsion destabilization was analyzed using backscattering (BS) curves for different sample heights. The sample with a height of 0 mm corresponds to the bottom of the cuvette. The Turboscan stability index (TSI) was used to determine the stability of the whole dispersion system [36,43].

### 2.4. Determination of Wound Healing Potential of the Creams

Male Sprague–Dawley rats weighing 263–299 g were used for in vivo wound healing studies. The animals were maintained on a standard pellet diet and water ad libitum throughout the experiments. The animals were divided into five groups (six animals placed in each group): Control group (untreated group, only handled the same way to see the effect of experimental conditions), Placebo group (treated with PC), Reference group (treated with Madecassol^®^ (M) which contains 10 mg/1 g *Centella asiatica* extract), Complex group (treated with CC) and Levant storax group treated with LSC. This study was approved by the Experimental Animals Ethics Committee at Yeditepe University Medical Faculty (Ethical committee decision no: 129).

#### 2.4.1. Excisional Wound Model

The wound healing efficacy of the creams was evaluated in an in vivo excisional wound model utilizing rats. The performance of LSC and CC was compared against the commercial reference cream, Madecassol^®^ (M), as well as the PC. A negative control group of rats that received no treatment was also included. Wound contraction percentages, rates of full skin thickness development, and histopathological parameters were employed to assess the effectiveness of the different treatments [46,47].

**Figure 1:**
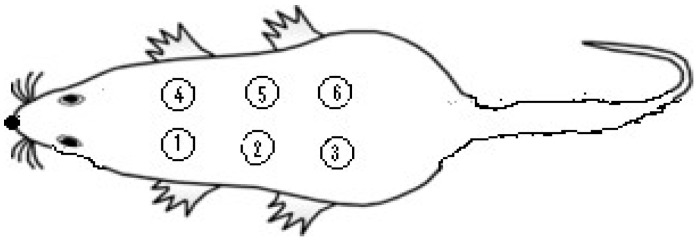
Template used for wound numbering, when excisional wounds were created at the back of the animals.

This wound model was commonly used to monitor wound contraction rate and wound closure time. Each group of animals (six animals in each group) was anaesthetized using Ketalar^®^ (Ketamine HCl 500 mg/mL; 100 mg/kg) and Rompun^®^ (Xylazine HCl 2%; 10 mg/kg). The hair on the back of the rats was depilated by shaving with a single-use razor, after which the shaved area was disinfected with 70% ethanol. The rat’s skin was punched using a 5 mm punch (Figure 1). The punching was repeated to create a total of six wounds on the back of each animal. After completing the excisional wounding, the wounds of the control group were left untreated. LSC, CC, M, and PC were applied topically once a day until the wounds of all animals in a group were completely healed, as summarized in Table 2. Treatment of the animals ended upon achieving complete healing in one of the groups. All animals in the LSC group demonstrated full recovery by the ninth day of treatment. Consequently, animals across all experimental groups were sacrıficed by ınjectıng a high dose of anesthetic. Tissue samples were collected for histopathological evaluation and for further analysis. Biopsy punches were used to isolate tissue samples from the healed skin of each animal, which were then fixed in 10% buffered formaldehyde for histopathological examination and to measure the full-thickness skin. Additionally, three intact tissue samples were taken from the surrounding area of the wounds using a biopsy punch to assess the full skin thickness and compare these with the healed skin thickness.

#### 2.4.2. Determination of Wound Contraction Rate

**Figure 2:**
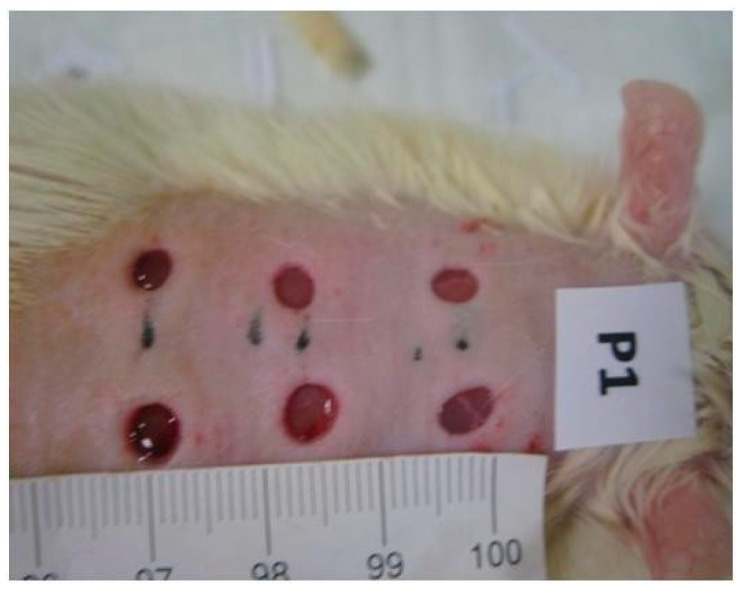
Images of one of the placebo group’s animals’ wound areas measured and monitored with a standard reference ruler and by a camera (Nikon^®^).

The progressive changes in the wound area were measured by means of photographs of the wounds taken by camera (Nikon^®^, Tokyo, Japan) every other day. To facilitate calibration, a standard reference ruler was placed near the wound when the pictures were taken (Figure 2). All wounds were also photographed with a D Lite Analog Microscope on the first and last day of the study, again in the presence of a standard reference ruler. The photographs were used to compute the wound contraction rates. Image J 1.44 software version 1.44 was used for wound area calculations [48,49]. The wound contraction rates were calculated as the percentage of reduction in the wound area over time. The wound contraction rates were statistically compared between the groups to ensure significance [48,49].

#### 2.4.3. Histopathological Evaluations

In the histopathological studies, after the routine fixation procedures, the tissues were embedded in paraffin wax. Serial sections of paraffin-embedded tissues of 5 μm thickness were cut. To assess vascularization, active and chronic inflammation, fibroblastic activity, fibrosis, and hair follicle development processes, hematoxylin and eosinstained preparations were examined under a Leica CTR 6000 microscope fitted with a DFC 350 FXR2 camera. To evaluate histopathological parameters such as active and chronic inflammation, neovascularization, fibrosis, and fibroblastic activity levels, and to determine the healing phase of each group. All findings were scored from 0 to 3, which signified nil (0), mild (1), moderate (2), and severe (3) [50].

#### 2.4.4. Evaluation of Full Skin Thickness

The stained, healed wounds and sections of intact tissue were photographed in the presence of a standard reference ruler using a D Lite Analog Microscope, which was plugged into a computer to record the pictures. The skin thickness of the wounds from healed and intact nearby tissue was measured using Image J software, and the results were compared statistically for significance.

#### 2.4.5. Analysis of Data

One-way analysis of variance (ANOVA) was used to statistically analyze wound contraction rates and full skin thickness development rates. Data obtained from the histopathological evaluations was statistically analyzed using Kruskal–Wallis with the Mann–Whitney U tests. *p* < 0.05 was considered significant. The SPSS (Statistics 29) program was used for statistical data analysis.

## 3. Results

### 3.1. Development of the Formulations and Stability Results

As explained in Section 2.2.1, cream formulations were prepared. Table 3 summarizes the composition of the formulations, and Table 4 summarizes the characterization results of the creams.

During the stability studies, no changes were observed in the organoleptic properties (appearance, color, odor, phase separation), as summarized in Table 5, and in the properties as summarized in Table 4 (pH, viscosity, conductivity, and zeta potential). The Microscopic examinations and particle size measurements (Table 6, Table 7 and Table 8), as well as the Turbiscan™ measurements, supported the findings that all formulations showed good stability during the experiments. There was no significant globule size change (coalescence) or phase separation (sedimentation, creaming) observed.

### 3.2. Wound Healing Efficacy of the Creams

#### 3.2.1. Wound Contraction Rate

**Figure 3:**
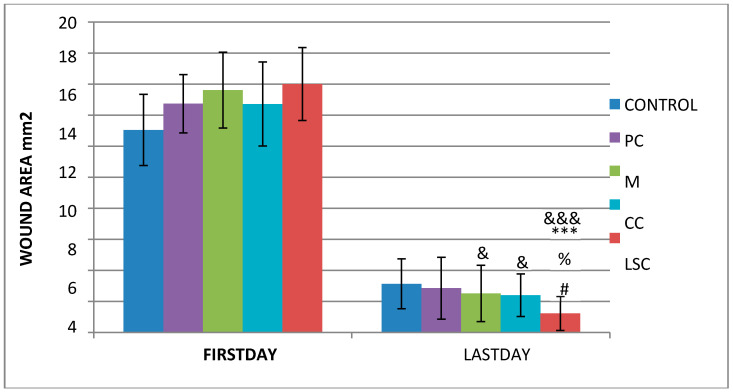
Wound contraction performance according to photographs taken by DLite Analog Microscope LSG ‘&’; compared to the control group, ‘*’; compared to the placebo group, ‘%’; compared to the reference group, ‘#’; compared to the complex group. &, #, % *p* < 0.05 ***, &&& *p* < 0.001.

As detailed in Section 2.4.1 and Section 2.4.2, and excisional wound model was employed on rats to assess the wound healing potential of the creams. One-way ANOVA with Tukey post-hoc tests was used to analyze differences between groups at different time intervals (producing significant results obtained by both photography techniques (Figure 2) are summarized in Table 9 and Table 10. The results clearly showed that the LSC group showed the best healing performance among all groups, while the CC and M groups demonstrated better wound healing activities than the control group. Figure 3 summarizes the wound area measurements of all treatment groups on the first day and the last day of the treatments. Healing potential of LSC was the highest compared to all groups (*p* < 0001).

#### 3.2.2. Full Skin Thickness

The other parameter used to monitor the healing effectiveness/efficacy was the measurement of the treated skin thickness and comparison of it with intact skin thickness. The results are shown in Table 11, and the differences were statistically analyzed. One-way ANOVA with Tukey post-hoc tests was used to analyze differences between the groups; LSC showed significantly increased wound healing activity when compared to the C, PC, and M groups. The CC group also demonstrated significantly better results when measured against the PC group.

**Figure 4:**
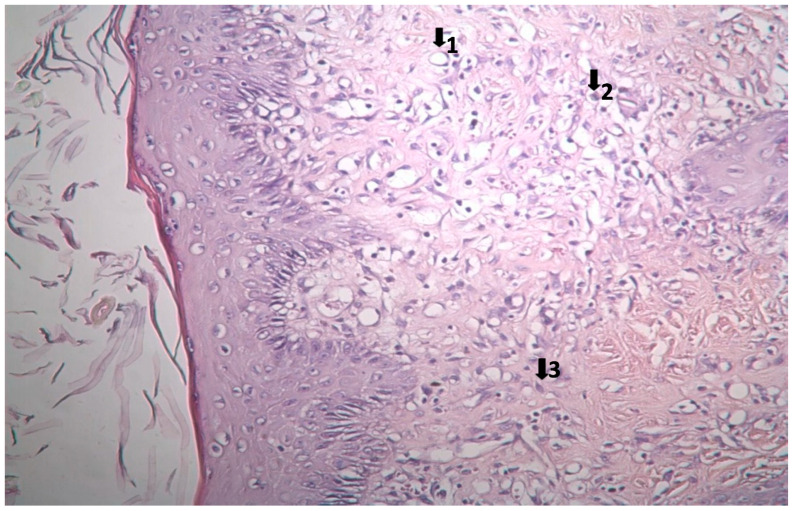
Histopathological view of wound healing in the control group (C). The skin sections show the hematoxylin and eosin stained epidermis and dermis. Arrows indicate events during wound healing; 1, neovascularization; 2, mononuclear cell; 3, fibroblast.

The results of histopathological studies, as the mean scores of the parameters investigated for all groups, are shown in Table 12. Examples of microscope slides of histopathological views of wound healing for each treatment group are presented in Figure 4. All data obtained from histopathologic observation was computed and statistically analyzed using the Kruskal–Wallis test for all histopathologic parameters and the Mann–Whitney U test for variations between paired groups. In both analyses, significant differences were found when it came to active and chronic inflammation, vascularization level, fibrosis, and fibroblastic activities. The LSC group scored highest for all healing indicators.

### 3.3. Histopathological Results

**Figure 5:**
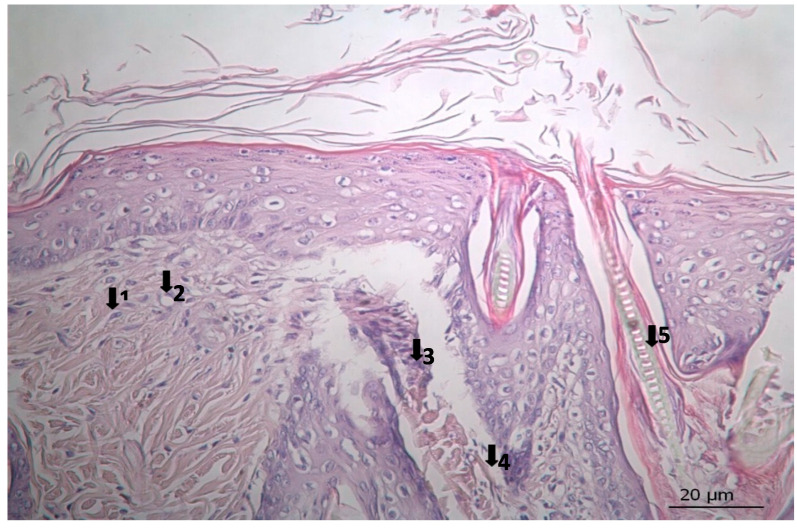
Histopathological view of wound healing in the placebo group (P). The skin section shows the hematoxylin and eosin stained epidermis and dermis. Arrows indicate events during wound healing: 1, fibroblast; 2, neovascularization; 3, neutrophil; 4, congestion; 5, hair follicle.

**Figure 6:**
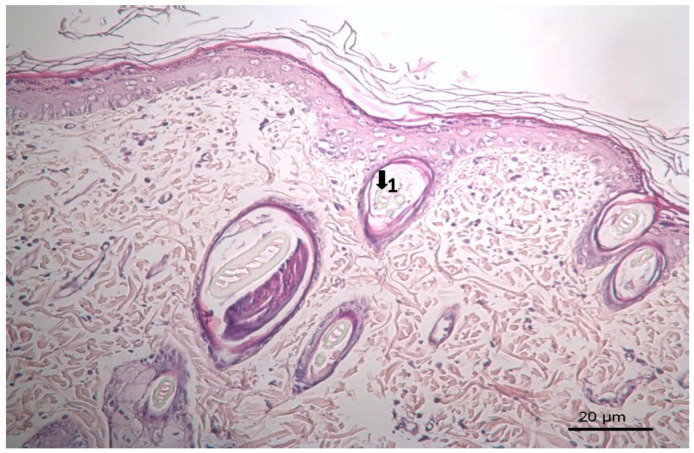
Histopathological view of wound healing in the reference group (M). The skin section shows the hematoxylin and eosin stained epidermis and dermis. Arrows indicate events during wound healing; 1, hair follicle.

**Figure 7:**
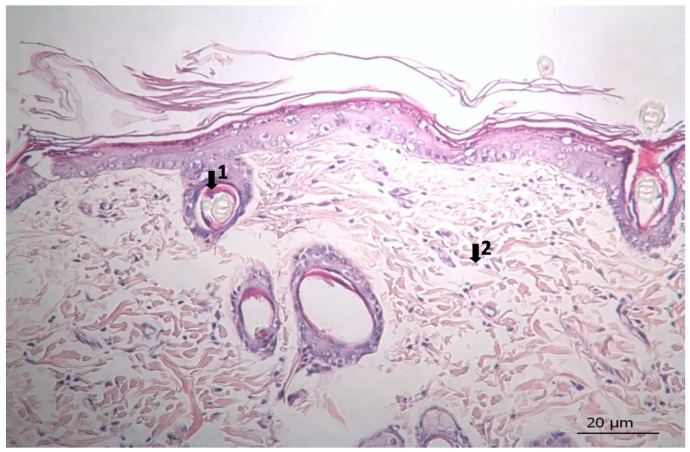
Histopathological view of wound healing in the complex group. The skin section shows the hematoxylin and eosin stained epidermis and dermis. Arrows indicate events during wound healing; 1, hair follicle; 2, collagen fiber.

**Figure 8:**
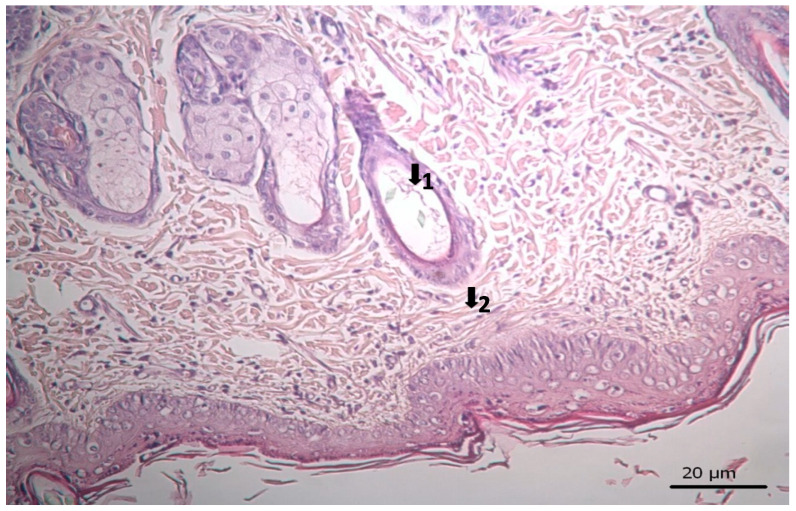
Histopathological view of wound healing in the Levant storax group. The skin section shows the hematoxylin and eosin stained epidermis and dermis. Arrows indicate events during wound healing; 1, hair follicle; 2, collagen fiber.

The results of histopathological observations are summarized in Table 12. Histopathological views of wound healing in the different groups are shown in Figure 4, Figure 5, Figure 6, Figure 7, and Figure 8 for C, P, M, CC, and LSC respectively. All results obtained from histopathologic observations were analyzed using the Kruskal–Wallis test for all histopathological parameters and Mann–Whitney U test for variations between two groups. Given all these results, the differences were significant in the wound healing phases of active and chronic inflammation, vascularization level, fibrosis, and fibroblastic activity. LSC was the best performing group in all favourable scores for the healing indicators.

## 4. Discussion

This study aimed to develop two stable emulsion-based creams for wound healing applications and to evaluate their effects using an in vivo animal model. Initial formulation efforts resulted in developing a placebo cream base by preparing nine different variants with differing excipients and varying amounts of them to ensure adequate viscosity, spreadability, acceptable after-feel on application on the skin, and stability. The formulation coded as P009 was selected as the optimal placebo base, which provided essential features for maintaining wound moisture and adherence to skin (Table 3). Subsequently, two active creams, CC and LSC, were formulated by incorporating specific herbal extracts into this placebo base. Functional actives in the CC included Calendula oil, St. John’s wort extract, aescin, freeze-dried Aloe vera (L.) Burm.f. leaf juice, and allantoin, chosen based on literature and supplier guidelines [11,12,13,14,15,16,17,18]. LSC contained only balsam of oriental sweet gum as the functional active ingredient. Similarly, based on published studies, especially for its antibacterial activity, balsam of *Liquidambar orientalis* was used at concentrations around 10% [21,22,23].

Characterization of the P, CC, and LSC encompassed organoleptic properties, pH, viscosity, conductivity, zeta potential, and particle size distribution analyses. Many researchers have shown that herbal extracts may act as electrolytes and negatively affect the stability of herbal creams, and they often cause separation of the phases in a short time [15,51]. Despite the negative effects of the herbal extracts, neither CC nor LSC showed stability problems such as phase separation or coalescence (Table 5). Inadequate moisture in the wound bed would impede the healing process; therefore, wound care products are expected to cover the wound and remain in place for at least some time, like a wound dressing product [34,52]. In this study, the viscosity of the formulations was regarded as an important parameter during the initial phase of formulation development. Among the formulations, LSC exhibited the highest viscosity due to its viscous balsam component; however, it was easily spread on the skin. Active ingredient incorporation increased conductivity values, consistent with electrolyte presence, confirming all formulations were oil-in-water emulsions. The formulations maintained acidic pH (Table 4), which fell within the physiological skin pH range, which is also suitable to inhibit microbial growth in the cream [45] and support the wound healing process [53]. Stability assessments over two months, including centrifugation, freeze-thaw cycles, particle size distribution measurements, and Turbiscan™ light scattering analysis, demonstrated no phase separation, significant particle size changes, or organoleptic deterioration (Table 5, Table 6, Table 7 and Table 8), indicating good stability at the duration of this study. Zeta potential measurements have also been widely used to quantify the magnitude of the electrical charge at the double layer, and several studies have been conducted to illustrate the relationship between the magnitude of the zeta potential and the stability of the creams. Based on the data and general observations, zeta potential values close to ±30 mV are regarded as having good stability [36,38,39,44]. In this study, all cream formulations exhibited zeta potential values around −30 mV, indicating good stability. These values remained consistent throughout the study period, suggesting that the emulsions maintained electrostatic stability. This stability is likely due to the presence of surfactants (cetearyl olivate and sorbitan olivate), which form lamellar liquid crystalline structures. These emulsifiers not only contribute to the physical stability of the formulations but also provide protective effects by preventing moisture loss, enhancing skin softness, and improving spreadability [54].

Microbiological tests conducted on the creams developed confirmed the efficacy of the preservative system used in the formulations (Uniphen P 23), ensuring microbial integrity during storage.

The wound healing process is an active, dynamic phenomenon initiated following disruption of tissue integrity and may extend over days, months, or even years, involving intertwined and complex effects that resist precise temporal delineation [1,2,3,4,5]. Normal wound healing includes hemostatic/inflammatory, proliferative/cellular, and maturation/remodeling phases [1,2,3,4,5,6,7,8]. Various topical and systemic agents have been employed for the treatment, and the primary goal is to achieve optimal cosmetic and functional outcomes by modulating factors involved in healing—such as inflammatory cells, platelets, mediators, and extracellular matrix—thus shortening healing time and ideally minimizing scar formation [1,2,3,4,5,6,7,8].

An excisional wound model in rats was employed to evaluate the wound healing potential of the formulations developed for this study. Wound area changes are presented in Table 9 and Table 10, indicating that the higher viscosity and adhesive properties of the LSC, attributed to the balsam’s physical characteristics, facilitate enhanced wound coverage and moisture retention. This protective effect likely minimized desiccation, eschar formation, and promoted epithelialization, resulting in significantly improved wound contraction rates (Figure 3). Additionally, antimicrobial constituents within the balsam [21,22,24,25,27,28] likely suppressed microbial colonization, further supporting wound repair. Consequently, LSC demonstrated the most pronounced wound contraction among all groups (Figure 3). While antimicrobial and moisturizing effects account for some of these observations, additional mechanisms have been reported on its anti-ulcerogenic effects [23], wound healing potential in traditional use [23,24,25,26,28,55], effect on mycobacteria [27], and effects on cancer cells [30,31,32]. It is also important to note that evaluation of Styrax liquidus in terms of its genotoxicity and mutagenicity indicated that it is safe up to 2000 mg/kg body weight, and Styrax is not genotoxic in mammalian bone marrow chromosome aberration test in vivo; the IC_50_ values of Styrax and its ethanolic extract were found to be 50.22 ± 1.80 and 59.69 ± 11.77 μg/mL, respectively [29].

The CC contained well-established actives such as aescin, Aloe vera, Allantoin, Calendula oil, and *Hypericum perforatum* extract. These herbal remedies are known for their fibroblast stimulation, anti-inflammatory, and anti-edematous effects [10,11,12,13,14,15,17,18], which translates to statistically significant improvements over C and PC groups in terms of wound contraction (Figure 3) and progressive changes in wound area measurements (Table 9 and Table 10). The results regarding full skin thickness regeneration are detailed in Table 11. The LSC group exhibited superior effects on skin thickness restoration, followed by the CC, which outperformed the P and C (control) groups, likely due to its fibroblast-stimulating constituents. Wound healing proceeds through hemostasis, inflammation, proliferation, and remodeling phases. Histopathological evaluations utilized cellular markers to delineate these stages: neutrophils and mononuclear cells as indicators of inflammation [56], while the recovery phase of wound healing is marked by fibrosis and hair follicle formation, decreased neovascularization, and disappearance of neutrophils and mononuclear cells [56,57,58]. In histopathological investigations of this study, neutrophils and mononuclear cells are used as indicators of the inflammation phase [56]. Histopathological views presented in Figure 4, Figure 5, Figure 6, Figure 7 and Figure 8 and a summary of results of the seen histopathological indicators (Table 12) showed minimal active inflammation, evidenced by low neutrophil presence across all groups. Chronic inflammation, defined by mononuclear cell infiltration [50,59,60], was absent in the LSC and CC groups but notable in the C and PC groups (Table 12), which underscored the anti-inflammatory properties of active ingredients. On the other hand, the proliferation phase is assessed by fibroblastic activity and neovascularization [56,57,58]. Fibroblast activity, critical for collagen synthesis and extracellular matrix production {5}, was also highest in the LSC group, aligning with its enhanced healing capacity. Neovascularization, essential for oxygenation and nutrient supply during new tissue formation [61,62] was significantly improved in LSC and CC groups compared with the other groups, supporting the multifaceted roles of the active contents in accelerating wound healing (Table 12). Fibrosis, defined as excessive collagen deposition leading to scar formation, was reduced in P and LSC groups, indicating less aberrant repair [60,63,64], and hair follicle neogenesis, which is an important parameter of advanced healing and regeneration and correlates with remodeling [60,64,65,66]. This was most pronounced in the LSC-treated wounds (Table 12, Figure 8). At the designated time point (day 9), wounds treated with LSC and CC had transitioned into the remodeling phase, while C- and PC-treated wounds remained in the proliferation phase. The M group was nearing remodeling (Figure 6). Collectively, these findings substantiate the superior wound healing efficacy of LSC and CC relative to others used as controls and comparisons.

The histopathological examination on the day of sacrifice demonstrated that the wounds which were treated with LSC and CC were in the recovery phase, while the M group was at the beginning of the recovery phase, and the control and the PC groups were still in the proliferation phase.

## 5. Conclusions

Two stable cream formulations were successfully developed as wound healing creams, and their efficacy was demonstrated using an in vivo animal wound healing model. Wound care studies indicated that LSC-treated rats showed the best healing rates compared to control, M, CC, and PC groups, whereas CC-treated animals showed better healing rates than the control and PC groups. No significant differences were found between the CC and M groups. It can be concluded that both LSC and CC can be successfully used as wound care products.

## Figures and Tables

**Table 1:** The ingredients used in the PC, LSC, and CC.

Trade Name	Manufacturer
Cetiol^®^ SB 45	BASF SE (67056 Ludwigshafen, Rhein, Germany)
Fitoderm^®^	BASF SE (67056 Ludwigshafen, Rhein, Germany)
Olivem^®^ 1000 Crystal Skin	Hallstar Darien Manufacturing (Darien, IL 60561, USA)
Lanette^®^ O	BASF SE (67056 Ludwigshafen, Rhein, Germany)
Myritol^®^318	BASF SE (67056 Ludwigshafen, Rhein, Germany)
Vaseline^®^	Sigma Aldrich (St Louis, MO 63103, USA,)
Pricerine™ 9091	Croda International PLC (East Yorkshire DN149AA, UK)
EDTA	Merck KGaA, (Darmstad, Germany)
Horse Chestnut Ext. 90%	Sami-Sabinsa Group Ltd. (Bengalore 560058, India)
Calendula Oil	Provital S.A. (Barcelona, Spain)
Aloe vera Powder 100X	Terry Laboratories LLC (Melbourne, FL 32904, USA)
Allantoin	Sigma-Aldrich (St Louis, MO 63103, USA)
Phytami^®^ St. John’s Wort	Alban Muller by Croda International PLC (East Yorkshire, DN149AA, UK)
Uniphen P 23	Induchem Components Ltd. (Cork P43D959, Ireland)

**Table 2:** Animal groups and summary of their wound care plans.

Animal Group Name	Number of Animals	Feeding Method	Wound Care Plan
Control group (C)	6	Standard pellet diet and water ad libitum	Untreated
PC group	6	Standard pellet diet and water ad libitum	PC applied topically once a day
M group	6	Standard pellet diet and water ad libitum	M applied topically once daily
CC group	6	Standard pellet diet and water ad libitum	CC applied topically once a day
LSC group	6	Standard pellet diet and water ad libitum	Levant storax cream applied once daily

**Table 3:** The content of formulations.

Ingredients (INCI Names)	Trade Names	PC (*w*/*w*)	CC (*w*/*w*)	LSC (*w*/*w*)
Shea butter	Cetiol^®^ SB 45	2.80	2.80	2.80
Squalene	Fitoderm^®^	2.80	2.80	2.80
Cetearyl olivate and sorbitan olivate	Olivem^®^ 1000 Crystal Skin	6.00	6.00	6.00
Cetostearyl alcohol	LANETTE^®^ O	2.00	2.00	2.00
Caprylic/capric triglyceride	MYRITOL^®^318	7.00	7.00	7.00
Petroleum jelly	VASELINE^®^	7.00	7.00	7.00
Glycerine	PRICERINE™	14.00	14.00	14.00
Ethylenediaminetetraacetic acid	EDTA	0.10	0.10	0.10
Escin 90%	Horsechestnut	----	xxx	--------
Calendula Oil	Calendula Oil	----	xxx	--------
*Aloe Barbadensis* leaf juice and maltodextrin	Terry-Spray *Aloe vera* Powder 100X	--------	xxx	--------
Allantoin	Allantoin	----	xxxxx	--------
*Glycerine, Water, Hypericum perforatum* extract	Phytami^®^ St. John’s wort	-----	xxx	--------
Balsam of oriental sweet gum	Oriental sweet gum	-----	----	xxxxxx
Methylparaben, Ethyl paraben, Propylparaben, Butylparaben, Isobutyl paraben, Phenoxyethanol	UNIPHEN P23	0.80	0.80	0.80
Deionized Water qs		100	100	100

**Table 4:** Summary of the characterization studies of the creams.

Name of Cream Formulation	pH	Conductivity (μs/cm)	Viscosity (kcps)	Zeta Potential (mV)
PC	4.85 ± 0.16	52.10 ± 3.54	33.94 ± 0.27	41.65 ± 1.63
CC	4.18 ± 0.05	111.00 ± 1.41	39.23 ±2.27	36.10 ± 1.13
LSC	4.47 ± 0.42	70.88 ± 4.07	71.45 ± 0.93	37.70 ± 0.60

**Table 5:** Summary of the stability test results, in terms of organoleptic properties. ‘√’: no change was observed in A: appearance, C: color, O: odor, PS: phase separation, ~: not tested.

		T0				1th Week				4th Week				8th Week			
		A	C	O	PS	A	C	O	PS	A	C	O	PS	A	C	O	PS
+4–8 °C	PC	√	√	√	√	√	√	√	√	√	√	√	√	√	√	√	√
	CC	√	√	√	√	√	√	√	√	√	√	√	√	√	√	√	√
	LSC	√	√	√	√	√	√	√	√	√	√	√	√	√	√	√	√
25 °C ± 2 °C 6O ± 5%RH	PC	√	√	√	√	√	√	√	√	√	√	√	√	√	√	√	√
	CC	√	√	√	√	√	√	√	√	√	√	√	√	√	√	√	√
	LSC	√	√	√	√	√	√	√	√	√	√	√	√	√	√	√	√
Room Temperature	PC	√	√	√	√	~	~	~	~	~	~	~	~	√	√	√	√
	CC	√	√	√	√	~	~	~	~	~	~	~	~	√	√	√	√
	LSC	√	√	√	√	~	~	~	~	~	~	~	~	√	√	√	√

**Table 6:** Calculated arithmetic mean diameters of particle size for placebo cream batches at different conditions and time intervals. PC; Placebo, Cream B1, B2, and B3 represent batch numbers.

Sampling Time (Weeks)	+4–8 °C			+25 °C ± 2 °C 65% RH			Room Temperature		
	PCB1	PCB2	PCB3	PCB1	PCB2	PCB3	PCB1	PCB2	PCB3
	Arithmetic Mean Diameter (μm)								
0	0.04	0.02	0.03	0.04	0.02	0.03	0.04	0.03	0.03
4	0.10	0.10	0.11	0.13	0.11	0.15	0.17	0.15	0.13
8	0.21	0.10	0.14	0.16	0.20	0.21	0.16	0.17	0.13

**Table 7:** Calculated arithmetic mean diameters of particle size for complex cream batches in different conditions and time intervals. CC: Complex Cream, Cream B1, B2, and B3 represent batch numbers.

Sampling Time (Weeks)	+4–8 °C			+25 °C ± 2 °C 65% RH			Room Temperature		
	CCB1	CCB2	CCB3	CCB1	CCCB2	CCB3	CCB1	CCB2	CCB3
	Arithmetic Mean Diameter (μm)								
0	0.19	0.06	0.22	0.19	0.06	0.22	0.19	0.06	0.22
4	0.18	0.17	0.18	0.17	0.15	0.17	0.17	0.15	0.17
8	0.17	0.14	0.24	0.16	0.11	0.18	0.18	0.16	0.18

**Table 8:** Calculated arithmetic mean diameters of particle size for Levant storax cream batches at different conditions and time intervals. LSC; Levant storax cream, Cream B1, B2, and B3 represent batch numbers.

Sampling Time (Weeks)	+4–8 °C			+25 °C ± 2 °C 65% RH			Room Temperature		
	LSCB1	LSCB2	LSCB3	LSCB1	LSCB2	LSCB3	LSCB1	LSCB2	LSCB3
	Arithmetic Mean Diameter (μm)								
0	0.12	0.17	0.24	0.12	0.17	0.24	0.19	0.12	0.17
4	0.15	0.16	0.19	0.21	0.21	0.30			
8	0.25	0.24	0.24	0.20	0.17	0.22	0.18	0.20	0.17

**Table 9:** Progressive changes in wound area of all groups monitored by camera (Nikon^®^).

Day	C Group	PC Group	M Group	CC Group	LSC Group
WOUND AREA (mm^2^)					
0	14.08 ± 2.02	13.95 ±1.39	14.44 ± 2.00	14.54 ± 1.69	14.48 ± 1.59
2	12.30 ± 1.65	11.77 ± 1.96	12.25 ± 2.15	12.43 ± 2.65	11.80 ± 2.10
4	10.83 ± 1.86	10.01 ± 1.94	10.49 ± 2.04	10.65 ± 2.45	10.01 ± 2.26
6	7.38 ± 2.49	7.16 ± 1.86	6.27 ± 3.12	6.63 ± 2.19	5.98 ± 2.79
9	2.93 ± 1.51	2.55 ± 2.84	2.00 ± 1.61	1.81 ± 1.67	1.50 ± 1.51

Values are expressed as mean ± SD, N: ~30.

**Table 10:** Progressive changes in wound area of all groups monitored by DLite Analog Microscope on days 0 and 9.

Day	C Group	PC Group	M Group	CC Group	LSC Group
	WOUND AREA (mm^2^)				
0	13.05 ± 2.29	14.74 ± 1.88	15.62 ± 2.44	14.72 ± 2.71	16.00 ± 2.35
9	3.13 ± 1.61	2.85 ± 1.99	2 ± 1.81	2.40 ± 1.37	1.22 ± 1.10

Values are expressed as mean ± SD, N: ~30.

**Table 11:** Ninth day skin thickness results of intact and treated skin tissue.

C	PC	M	CC	LSC
Thickness of wounds				
75.26 ± 9.64	63.41 ± 11.03	76.18 ± 10.98	80.55 ± 5.64	93.52 ± 15.52

**Table 12:** Histopathology results; ‘&’; compared with the control group (C), ‘*’compared with the PC group, ‘%’; CC with the M group, ‘#’; compared with the CC group ^a^. ^a^ Hematoxylin and eosin (HE) stained sections were scored as nil (0), mild (1), moderate (2) and severe (3) for dermal and epidermal re-modelling., #, &, *,% *p* < 0.05; %%, ** *p* < 0.01; ***, &&&, %%%; *p* < 0.001.

Group Name	Active Inflammation	Chronic Inflammation	Fibroblastic Activity	Neovascularization	Fibrosis	Hair Follicle Formation	Healing Phase
Control	1.00 ± 0.47	1.28 ± 0.38	1.83 ± 0.54	1.78 ± 0.61	0.56 ± 0.46	0.47 ± 0.27	2.44 ± 0.46
Placebo	0.60 ± 0.38	1.33 ± 0.31	1.77 ± 0.56	1.60 ± 0.38	0.13 ± 0.18	0.53 ± 0.18	2.07 ± 0.54
Reference	0.39 ± 0.49	0.78 ± 0.49	1.19 ± 0.87	1.11 ± 0.85	0.67 ± 0.29	0.33 ± 0.30	2.67 ± 0.42 *
Complex	0.22 ± 0.40	0.52 ± 0.37 &*	0.77 ± 0.20	0.52 ± 0.40 &&&**	0.42 ± 0.36	0.56 ± 0.17	3.00 ± 0.00 &***
Levant Storax	0.11 ± 0.27	0.36 ± 0.12 &&&***	0.13 ± 0.19 &&&*** %%#	0.22 ± 0.13 &&&***	0.27± 0.13 %	0.94 ± 0.13 &%%%	3.00 ± 0.00 &***

## Data Availability

The original contributions presented in this study are included in the article. Further inquiries can be directed to the corresponding author.
